# Maternal birth preparedness and complication readiness in the Greater Accra region of Ghana: a cross-sectional study of two urban health facilities

**DOI:** 10.1186/s12884-020-03263-6

**Published:** 2020-09-25

**Authors:** Cynthia Klobodu, Brandy-Joe Milliron, Kofi Agyabeng, Patricia Akweongo, Augustine Adomah-Afari

**Affiliations:** 1 Department of Health Policy, Planning and Management, School of Public Health, College of Health Sciences, University of Ghana – Legon, P.O. Box LG 13, University of Ghana, Legon, Ghana; 2 Department of Nutrition Sciences, Centre for Family Intervention Science, College of Nursing and Health Professions, Drexel University, 1601 Cherry Street. Suite 383, Philadelphia, PA 19102 USA; 3 Ministry of Education, National Inspectorate Board, Private Mail Bag 18, Ministries Post Office, Ameda Street, Yooyi Ln, Accra, Ghana

**Keywords:** Birth preparedness and complication readiness, Skilled birth attendance, Safe motherhood initiative, Antenatal clinic, Postnatal clinic

## Abstract

**Background:**

High maternal mortality ratios remain a critical public health concern in Ghana. Birth preparedness and complication readiness (BP/CR), which is a component of focused antenatal care, is a safe motherhood strategy intended to promote skilled birth attendance by helping women and their families plan for pregnancy and childbirth, thereby reducing maternal mortality. The objective of this study was to determine the level of BP/CR and to assess factors associated with maternal BP/CR in the Greater Accra Region of Ghana.

**Method:**

A cross sectional descriptive quantitative study was carried out among 300 postnatal women attending the Adabraka Polyclinic and the Greater Accra Regional Hospital both within Accra, the capital city of Ghana. Data were collected with a structured questionnaire which assessed socio-demographic, health facility/provider and social support factors and their associations with BP/CR. Levels of BP/CR were assessed using validated tools. Data from 300 women were analyzed using STATA version 15.0. Logistic regression analysis was conducted to establish associations between BP/CR and socio-demographic, health facility/provider and social support factors.

**Results:**

Approximately 234 (78%) of the women were birth prepared. Strong predictors of BP/CR included having ≥4 antenatal clinic visits (aOR 2.63; 95% CI 1.03–6.73), being employed (aOR 4.07; 95% CI 1.49–11.11) and belonging to maternal health promoting clubs or groups during the antenatal period (aOR 3.00; 95% CI 1.07–8.40) .

**Conclusion:**

BP/CR is generally high among the study population. Predictors of BP/CR are multifactorial and found to cut across all aspects assessed in the study**.** Therefore, the creation of a BP/CR tool is recommended to routinely monitor trends in maternal birth preparedness in antenatal clinics. This may help to sustain and improve current levels and indicators of BP/CR.

## Background

Although Sustainable Development Goal 3 (SDG3) has a target of reducing the maternal mortality ratio (MMR) to less than 70 per 100,000 live births in 2030, the burden in Ghana remains high with a MMR of 310 per 100,000 live births [1]. This goal may not be reached with the annual reduction rate of 2.7% as opposed to the required estimate of 7.5% [1, 2].

Data from the Ghana Health Service (GHS) showed that the highest number of maternal deaths was reported in the Greater Accra Region in 2016 with 130 deaths, closely followed by the Eastern Region with a little under 100 deaths [3]. A high proportion of direct maternal deaths was due to obstetric hemorrhage, followed by hypertensive disorders of pregnancy [3].

Skilled birth attendance is one of the most important factors for reducing maternal mortality and birth preparedness and complication readiness (BP/CR) is an approach to promote this [4, 5]. The prevalence of skilled birth attendance was recorded as 56.2% nationwide and 59.4% for the Greater Accra Region in 2016 according to GHS whereas the Demographic and Health Survey (DHS) reported 73.7% nationwide and 92.5% for the Greater Accra Region in 2014 [3, 5]. GHS attributes its lower numbers to lack of a centralized data collection system to capture births from private facilities and maternity homes [3].

The World Health Organisation (WHO) recommends that every pregnant woman should have a written BP/CR plan, discussed with a skilled birth attendant during every antenatal visit or at least one month prior to birth [6]. Antenatal clinics in Ghana are run by midwives, nurses and doctors and follow WHO’s focused antenatal care (FANC) approach [7]. FANC is a continuum of care which emphasizes evidence-based interventions including BP/CR for all women, in addition to providing care and support for women and their families [8].

Levels of BP/CR vary across the literature and are generally low [9]. Socio-demographic characteristics, including age, parity and education as well as socio-economic factors, such as income, have been shown to influence BP/CR across different socio-cultural settings. Social support factors, such as spousal, family and community support, also appear to influence BP/CR. Furthermore, definitions of BP/CR vary in the literature, which may account for inconsistencies between different studies [10]. The WHO’s BP/CR plan involves identifying: 1. place of birth; 2. preferred skilled birth attendant; 3. location of an appropriate facility for birth in close proximity; 4. funds for any expenses related to birth and emergencies; 5. a labor and birth companion; 6. care support at home while the woman is away; 7. transport to a health facility for birth 8. transport in case of obstetric emergencies and 9. compatible blood donors when needed [6]. The Johns Hopkins Program for International Education in Gynaecology and Obstetrics (JHPIEGO)‘s package provides well described roles for all stakeholders, including community members and policy makers, thereby making the plan more comprehensive [11].

Certain countries, such as Nepal, have instituted birth preparedness cards, also known as ‘key chains,’ with messages on birth preparedness [12]. Tanzania, Ethiopia, Uganda, Eritrea and Kenya included home visits by volunteers to educate families on BP/CR; training of health workers to provide information and support on BP/CR; provision of visual aids and educational materials on BP/CR; and community surveillance systems for pregnancies, which are all periodically assessed [10]. In Rwanda, Community Health Workers (CHWs) iterate BP/CR messages through mobile technology in the form of SMS alerts, in addition to home visits and community meetings [13].

BP/CR, however, is not routinely assessed in Ghana and researchers who have assessed BP/CR have not used consistent instruments. Little information on BP/CR is known in urban areas as most research has been conducted in rural Ghana [14–16]. This study intends to add knowledge on BP/CR using a systematic method. The objective is to determine levels of BP/CR and to assess factors associated with BP/CR in the Greater Accra Region of Ghana.

## Methods

### Study design, setting and population

A facility-based cross-sectional study was conducted in Adabraka Polyclinic, a primary care facility, and Greater Accra Regional Hospital a referral facility in Osu Klottey Sub-Metro (both facilities now lie within the Korle Klottey Municipal Assembly) of the Accra Metropolitan Assembly (AMA) between March 2019 and May 2019. The population of Osu Klottey Sub-Metro was 151,712 with 36,411 women of reproductive age and an expected number of pregnancies of 6068 in 2017. The Accra metropolis was chosen because it houses the capital city of the Greater Accra Region and Ghana.

### Sample size and sampling procedure

Sample size was determined using the single population proportion formula. A BP/CR level of 23.0% was considered from a previous study [15]. A sample size of 300 women was calculated by using 95% level of significance, 0.05 margin of error and a 10% non-response rate. A stratified random probability sampling method was used to recruit women from both facilities. Women were recruited to participate if they were between 18 and 49 years old, had given birth within the last six weeks, and attended postnatal clinics (PNC) in any of the two facilities.

Using PNC records of both facilities, Adabraka Polyclinic was estimated to have an average attendance of 50 women per week and a five weeks study period estimated an average of 250 women. Greater Accra Regional Hospital had an average attendance of 100 women per week and an estimated average of 500 women for the five weeks study duration. The required sample size was then proportionately allocated to the two health facilities with Adabraka Polyclinic having 100 women and Greater Accra Regional Hospital 200 women. Recruitement was done by mouth and through distribution of flyers in both facilities. Simple random samping was subsequently used in both facilities to enroll participants. A list of PNC attendees was entered in Microsoft Excel, unique numbers were assigned to them and the total number of women per day randomly chosen.

### Data collection

A structured questionnaire with four sections was designed for the purposes of this study. Section A included personal and sociodemographic characteristics, such as age, parity, marital status, income, religion, employment status and wealth index. Section B included data on facility and social support factors, such as proximity to the clinic, antenatal clinic (ANC) attendance during last pregnancy, place of birth (as a proxy for skilled birth attendance), and maternal health promoting clubs or groups membership. Section C included information regarding BP/CR using the Maternal and Child Health Records (MCHR) book of 2018 [17]. This book was developed by the Japanese International Cooperation Agency (JICA) in collaboration with the Ministry of Health and GHS in accordance with WHO’s recommendations on home-based records for maternal and newborn care, serving as a standard instruction guide and records’ book for women and health facilities across the country [18]. The questionnaire was either self- or interviewer-administered, depending on the educational background of the women.

### Operational definitions

Women were classified as birth prepared if they reported at least four out of seven factors in the MCHR book [17]. This scoring system has been employed by several other studies [2, 14, 15]. Those factors are: 1) making arrangements for transport; 2) making arrangements for helpers to take care at home while women are away during birth; 3) deciding where to give birth; 4) saving money for care and transport; 5) having a valid health insurance; 6) identifying a blood donor; and 7) having knowledge of 11 danger signs of pregnancy. These signs include: headache; swollen feet, arms and face; convulsive fits; breakage of bag of water before expected date of delivery (EDD); dizziness, difficulty in breathing and rapid heart beating; increase in body temperature; increase or decrease or no movement of baby; smelly or greenish water from birth canal; persistent vomiting; severe abdominal pain; and bleeding [17]. A score of ‘1 (= yes)’ was awarded for knowing all danger signs and a score of ‘0 (= No)’ for missing one danger sign. The decision to score + 1 point if the respondent knows all 11 danger signs is based on WHO’s recommendations as stated in the Counselling for Maternal and Newborn Health care handbook [19].

Due to uncertainties and bias in income reporting, wealth index was used as a proxy indicator. The asset-based wealth index provides a composite quantification of participants’ cumulative standard of living, using information gathered about ownership of certain selected assets. The Equity tool abridged wealth index for Ghana [20], comprising of a 13 household item list, was adapted to collect this data.

Using principal component analysis, an index was computed to put participants on a continuous scale of relative riches. Based on that, participants were then categorized into five wealth quintiles: the first 20th percentile group, representing the relatively poorest quintile of the participants and 5th quintile, representing the relatively richest participants.

### Data analysis

Descriptive statistics on categorical variables were reported in terms of frequencies and percentages. Bar charts were also used for pictorial illustrations. A multiple binary logistic regression model was used in determining predictors of BP/CR among women who recently had given birth. Results of the logistic regression model are reported as odds ratios with 95% confidence intervals. All statistical tests were done at 5% significance level.

## Results

### Socio-demographic characteristics of respondents

Out of the 300 included women 153 (51%) were ≤ 29 years, 230 (76.7%) were married or cohabiting and 225 (75%) were employed; 199 (66.3%) had between one and two children and 287 (95.6%) had some form of education with only 11 (3.7%) having no formal education (Table 1).

**Table 1:** Socio-demographic characteristics of women attending postnatal care

Variable	Frequency	Percentage
**Age group (Years)**		
≤ 29	153	51.0
30–39	114	38.0
40–49	31	10.3
Missing data	2	0.7
**Marital Status**		
Married/cohabiting	230	76.7
Single	62	20.7
Missing data	8	2.7
**Parity**		
1–2 children	199	66.3
More than 3 children	94	31.3
Missing data	7	2.3
**Religion**		
Christian	208	69.3
Non-Christian	89	29.7
Missing data	3	1.0
**Educational Level**		
No formal education	11	3.7
Primary	88	29.3
Secondary	82	27.3
Tertiary	117	39.0
Missing data	2	0.7
**Employment status**		
Unemployed	74	24.7
Employed	225	75.0
Missing data	1	0.3
**Wealth Index**		
Poorest	41	13.7
Poorer	41	13.7
Middle	42	14.0
Rich	40	13.3
Richer	41	13.7
Missing data	95	31.7
**Health Facility**		
APC	100	33.3
GARH	200	66.7

*APC* Adabraka Polyclinic, *GARH* Greater Accra Regional Hospital

### Health facility/provider and social support factors on BP/CR

Over half 172 (57.3%) of the women spent > 1 h to get to the nearest health facility. One hundred and eighty-four (61.3%) reported more than four antenatal visits during their last pregnancy (Table 2). About 295 (98.3%) reported that they had given birth in a health facility which was used as a proxy for skilled birth attendance, while only five women (1.7%) gave birth at home. The majority (281; 93.7%) of the respondents from both facilities reported having had some form of emotional and financial support during their last pregnancy. However, less than half (123; 41%) reported belonging to maternal health promoting groups or clubs during their last pregnancy (Table 2).

**Table 2:** Health facility/provider and social support factors on birth preparedness

Variable	Number	Percentage
**Average travel time to nearest health facility**		
< 1 h	172	57.3
≥ 1 h	127	42.3
Missing data	1	0.3
**ANC attendance for last pregnancy**		
< 4 visits	116	38.7
**≥** 4 visits	184	61.3
**Place of birth**		
Health Facility	295	98.3
Home	5	1.7
**Social support**		
received support	281	93.7
no support received	19	6.3
**Maternal health promoting club/ group membership**		
belonged to clubs/groups	123	41.0
did not belong to club/groups	177	59.0

*ANC* Antenatal Clinic

### Level and aspects of BP/CR

**Fig. 1:**
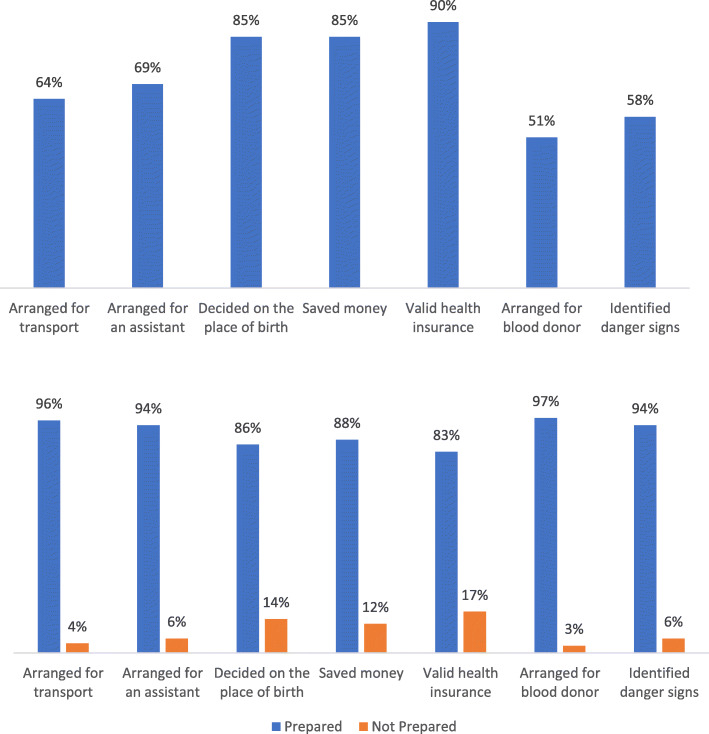
i: Aspects of BP/CR that were planned. Descriptive results presented as a percentage of participants prepared for each category, *n* = 300. Figure 1 ii: Aspects of BP/CR that were planned. Descriptive results presented as percentage of participants prepared for each category (n = 300), who were either birth prepared or not birth prepared

Of the 300 respondents, 234 (78%) were prepared for birth. A valid health insurance was held by 263 (90%), whereas only 148 (51%) had made arrangements for a blood donor (Fig. 1i). Two hundred and fifty-two (85%) decided on the place of birth and 248 (85%) also saved money for birth. Transport to the health facility was arranged by 187 (64%) and 202 (69%) arranged assistance to take care of the home during their absence. Only 171 (58%) were able to correctly identify all 11 danger signs in pregnancy (Fig. 1i). There were very significant differences in the proportions of the seven aspects of BP/CR between those who were considerd to be prepared and those not prepared (Fig. 1ii).

### Predictors of BP/CR among women who recently gave birth

From the multiple binary logistic regression model, ANC attendance for last pregnancy, employment status and maternal health promoting club or group membership were identified to be statistically significantl predictors of BP/CR (*p* < 0.05). Odds of being birth prepared was 2.6 times higher among women who had ≥4 ANC visits compared to those with < 4 ANC visits (aOR 2.63; 95%CI 1.03–6.73). With regards to employment status, mothers who were employed had ≥4 times higher odds of being birth prepared compared to unemployed women (aOR 4.07; 95% CI 1.49–11.11). Odds of being birth prepared among mothers belonging to maternal health promoting clubs or groups was 3 times higher compared to mothers who did not (aOR 3.00; 95% CI 1.07–8.40) (Table 3).

**Table 3:** Predictors of BP/CR among women who recently gave birth

	Unadjusted		Adjusted		
	cOR	95% CI	aOR	95% CI	
**Age Group**					
18–28	1.00		1.00		
29–39	3.33	1.79–6.21	2.15	0.79–5.91	
40–49	1.67	0.66–4.21	0.95	0.19–4.8	
**Marital Status**					
Married/Cohabiting	1.00		1.00		
Single/Divorced/Widowed	0.58	0.31–1.09	1.09	0.33–3.64	
**Parity**					
< 3	1.00		1.00		
≥3	2.54	1.29–5.03	2.21	0.61–8.02	
**Religion**					
Christian	1.00		1.00		
Non-Christian	0.79	0.44–1.43	1.60	0.55–4.68	
**Wealth Index**					
Poorest	1.00		1.00		
Poorer	2.01	0.7–5.78	0.96	0.25–3.69	
Middle	1.52	0.56–4.12	0.72	0.18–2.81	
Rich	2.34	0.78–7.03	3.67	0.68–19.85	
Richer	2.98	0.94–9.43	1.63	0.34–7.81	
**Average Travel Time to Nearest Health Facility**					
< 1 h	1.00		1.00		
≥ 1 h	1.14	0.65–1.99	1.69	0.64–4.43	
**ANC Attendance for last pregnancy**					
< 4 Visit	1.00		1.00		
≥ 4 Visits	3.48	1.97–6.14	2.63	1.03–6.73	
**Social Support**					
No support received	1.00		1.00		
Received support	7.21	2.71–19.17	4.24	0.87–20.55	
**Employment Status**					
Unemployed	1.00		1.00		
Employed	4.68	2.6–8.44	4.07	1.49–11.11	
**Educational Level**					
No formal education	0.22	0.06–0.79	0.04	0.00–0.78	
Primary	0.55	0.27–1.09	1.80	0.45–7.22	
Secondary	0.56	0.28–1.15	0.94	0.33–2.67	
Tertiary	1.00		1.00		
**Maternal Health Promoting Club/ Group Membership**					
Did not belong to club/groups	1.00		1.00		
Belonged to clubs/groups	1.52	0.86–2.69		3.00	1.07–8.4

*CI* Confidence interval, *cOR* Crude odds ratio, *aOR* Adjusted odds ratio

## Discussion

### Levels of BP/CR among women who recently delivered

We found 78% of the women to be birthprepared. This is consistent with a large community-based study in the Osogbo Metropolis of Southwest Nigeria, which found that 82.1% were birth prepared [21] and a study in an urban hospital in Hyderabad in India which found a 71.5% BP/CR level [22]. Many community and facility-based studies, however, have much lower BP/CR levels. For example, a multisite health facility based study in Kassena Nankana District and a community-based study in Sisala East District both of Northern Ghana reported that 16.2 and 23% of the women were birth prepared [14, 15]. A similar study in Tamale Teaching Hospital in the Northern Region of Ghana found BP/CR levels of 43.7%, while a mixed health facility and community-based study among teenage mothers in the Ledzorkuku Krowor Municipal Assembly in the Greater Accra Region reported 40.0% to be more birthprepared [16, 23]. Equally low rates have been reported in other countries with 20% BP/CR in a health facility-based study in Tharaka Nithi County in Kenya, 41.1% in Mizan-Tepi University Hospital in Southwest Ethiopia and 47.8% in a slum-based study with a functional maternal and child health program in Indore in India [24–26]. Discrepancies between our findings and these studies could be explained by socioeconomic factors. The studies in Kassena Nankana District, Sisala East District and Tamale Teaching Hospital were all conducted in northern Ghana, which is known to have wide socio-economic developmental gaps as compared with the southern part of the country [27]. In addition, the study among teenage mothers, although conducted in southern Ghana, had low levels of BP/CR because the mothers may not have been adequately empowered due to their status in society [23].

In our study, some aspects of BP/CR were more planned than others. The most prepared for was having a valid health insurance (90.4%), whereas the least was arranging for a blood donor (50.4%). Ghana’s National Health Insurance Scheme offers free maternal care and coverage involves four antenatal visits as well as the costs of skilled birth attendance [28]. This could have contributed to the high proportions of BP/CR in this aspect, although there may be some form of payments made during birth in health facilities. Similar to our findings, a study in Southwest Nigeria found that arranging for a blood donor was the least action women had undertaken towards BP/CR [21]. Generally, socio-cultural perceptions concerning blood donation and reception in Ghana hinder some people deciding to give or receive blood [29] despite high levels of iron deficiency anaemia, which is associated with post-partum hemorrhage especially amongst women of childbearing age [30]. The government must therefore work to improve national blood banking systems and promote blood donation efforts. Blood transfusion should also be discussed with pregnant women early on in the pregnancy to address misconceptions and alternative treatment methods [31].

Our study assessed knowledge on all 11 danger signs of pregnancy in the MCHR book according to WHO’s recommendations, and found only 58% of the women to be knowledgeable of all 11 signs. The leading causes of maternal mortality world wide include hemorrhage, hypertensive disorders and sepsis, and so it’s important for pregnant mothers to have adequate knowledge concerning signs associated with these conditions [32]. Studies show that women commonly miss danger signs associated with pre-eclampsia and eclampsia due to inadequate emphasis on all danger signs during antenatal visits [33]. However, certain danger signs may also be more clinically relevant than others depending on the stage of the pregnancy [33]. Therefore, more research is needed in this area to help prioritize danger signs during antenatal education. Having adequate knowledge of danger signs empowers women to identify and seek help early thereby improving maternal and fetal outcomes [34].

### Predictors of BP/CR

WHO currently recommends at least eight contacts with a health care provider during the antenatal period in order to improve maternal and perinatal outcomes [35]. Women who reported ≥4 antenatal visits were more likely to be birth prepared as compared with women with fewer visits, in agreement with studies in Southern Ethiopia, Kenya, Uganda and Ghana [10, 20, 33].

Similar to our findings, women with salaried work were 3.5 times more likely to be prepared for birth as compared with unemployed women in a study from Kenya [26]. Employed women are generally known to have more autonomy to make decisions concerning their health, thereby preventing delays in seeking healthcare [36–38]. In contrast, studies in South West Ethiopia and northern Ghana found no association between occupation and BP/CR [15, 39] . This could be attributed to the fact that most women in the Kassena Nankana District of Ghana were housewives and the Ethiopian government’s free provision of ambulance and other maternal and child health services [15, 39].

Maternity related community groups are shown to be generally supportive during pregnancy [40]. In India reported youth groups are undertaking activities to increase sensitization of maternal health problems and this improved maternal health outcomes [41]. Women who belonged to maternal health promoting clubs or groups during the antenatal period were found to be three times more likely to be birth prepared as compared with women without such clubs or groups. In our study, only 41% of the mothers belonged to such groups. Pregnant women may not always be aware of the supportive role played by the community in promoting BP/CR. This assertion is buttressed by a study in Nigeria where many women were not aware of existing community support mechanisms or programmes [42].

### Strengths and limitations

The criteria for BP/CR in this study were adapted from the MCHR 2018 book, which is the standard manual for focused antenatal care in all health institutions in Ghana. Therefore, the level of BP/CR could be presumed to be close to what actually exists amongst the study population. Additionally, the study was adequately powered with a large sample size of 300 participants to enable generalizability of the results. Furthermore, inclusion criteria for this study were limited to mothers who were within six weeks after birth to minimize recall bias.

High levels of BP/CR among the study population could be due to the fact that this was a health facility-based study and most women interviewed had been regular ANC attendants during their index pregnancy. Another limitation of the study was that most women had higher levels of education and this could have influenced the level of BP/CR, although education was not found to be a predictor of BP/CR. However, this educational level is more reflective of the level among women attending PNC in these urban facilities and not only those who decided to participate in the study.

## Conclusion

The level of BP/CR among study participants was generally high. Strong determinants of BP/CR included having ≥4 antenatal clinic visits, maternal employment and participation in maternal health promoting clubs or groups during the antenatal period.

Although BP/CR is one of the strategies employed by the Safe Motherhood Initiative to combat maternal mortality, there are no clearly defined criteria globally as to what it should entail [11]. Key definitions concerning BP/CR have not been agreed upon by experts, although various guidelines exist, and this makes assessment of interventions difficult [10]. Our study adds to the current literature on BP/CR and may guide future research and development of policy regarding BP/CR criteria that can be used to routinely assess BP/CR.

## Supplementary information

**Additional file 1: Appendix I**. Questionnaire.

## Data Availability

The datasets obtained from the current study will be made available by the corresponding author when requested.

## Abbreviations

ANC: Antenatal Clinic
aOR: Adjusted Odds Ratio
BP/CR: Birth Preparedness and Complication Readiness
CI: Confidence Interval
DHS: Demographic and Health Survey
EDD: Expected Date of Delivery
GHS: Ghana Health Service
JHPIEGO: Johns Hopkins Program for International Education in Gynaecology and Obstetrics
MCHR: Maternal and Child Health Records
PCA: Principal Component Analysis
PNC: Postnatal clinic
SDG: Sustainable Development Goal
WHO: World Health Organization
