# Supplementary material for: Maternal birth preparedness and complication readiness in the Greater Accra region of Ghana: a cross-sectional study of two urban health facilities
Source: BMC Pregnancy Childbirth. 2020 Sep 25;20:566. doi: 10.1186/s12884-020-03263-6 (PMC7519482; doi:10.1186/s12884-020-03263-6)
Supplement: Supplementary file 1 — Additional file 1: Appendix I. Questionnaire. [file 12884_2020_3263_MOESM1_ESM.pdf]

APPENDICES

APPENDIX I: QUESTIONNAIRE

Department of Health Policy, Planning and Management
School of Public Health
College of Health Sciences
University of Ghana

Introduction

This is a study to assess maternal birth preparedness towards delivery among postnatal women in the Greater Accra Region focusing on the Accra Metropolis. The results of this research will help inform policy makers on birth preparedness and factors associated with birth preparedness. Confidentiality of the answers given is assured. Thank you very much for your participation.

Instruction

Please answer by ticking your response or filling the space provided. Thank you.

Section A: Individual Patient (Socio-Demographics) Factors

Q1. Age (in years)…………..
Q2. Number of Children…………
Q3. Type of occupation
1. Housewife ( )
2. Civil Servant ( )
3. Self Employed ( )
4. Unemployed ( )

Q4.Religion
1. Christian ( )
2. Muslim ( )
3. Traditionalist ( )
4. Other, please specify…………………..

Q5. Estimated income per month …………….. /Estimated income per day…………….

Q6. Marital Status
1. Married ( )
2. Single ( )
3. Separated ( )
4. Cohabitating ( )
5. Widow ( )
6. Divorced ( )

Q7. Educational level
1. No formal education ( )
2. Primary school education ( )
3. Junior High School education (JHS) ( )
4. Senior High School education (SHS) ( )
5. Tertiary education ( )

Q8. How long does it take you to reach the nearest health facility to your house?
1. Less than 1 hour ( )
2. 1-2 hours ( )
3. 3-5 hours ( )
4. More than 5 hours

Q9. What is your common mode of transport to the health facility?
1. Private vehicle
2. Public transport
3. Motorbike
4. On foot
5. Others (specify)……………………………….

Q10. How many times did you attend antenatal clinic for your last pregnancy?
0. Never ( )
1. Once ( )
2. Twice ( )
3. Thrice ( )
4. Four times and above ( )

Section B: Health Facility and Provider factors
Q1. Where did you deliver your baby?
1. Health facility ( )
2. Home ( )

Q2. Which type of skilled attendants are available at the facility you delivered?
1. Nurses ( )
2. Midwives ( )
3. Doctors ( )

Q3. Are theatre services available at the facility?
0. No ( )
1. Yes ( )
2. Don’t know ( )

Q4. Do they run 24 hour services?

0. No ( )
1. Yes ( )
2. Don’t know ( )

Q5. Is there a functioning blood bank
0. No ( )
1. Yes ( )
2. Don’t know ( )

Q6. Were you referred to another facility?
1. Yes ( )
0. No ( )

Q7. If yes, how long did it take you to get there?
1. Less than 30 minutes ( )
2. Between 30 minutes to 1 hour ( )
3. More than 1 hour ( )

Q8. What can you say about the relationship the health workers had towards you at your place of delivery?

1. Very friendly ( )
2. Friendly ( )
3. Unfriendly ( )
4. Don’t know ( )

Section C: Social Support factors
Q1. Did you get support during pregnancy?
1. Yes ( )
0. No ( )

Q2. If yes from whom:
1. Husband ( )
2. Partner ( )
3. Friend ( )
4. Family ( ) specify………………..

Q3. How did they support you?
1. Financially ( )
2. Emotionally ( )

Q4. Are you satisfied with the support given you?
1. Highly satisfied ( )
2. Averagely satisfied ( )
3. Not satisfied ( )

4. Cannot tell ( )

Q5. Do you belong to mother clubs and groups which support each other?
1. Yes ( )
0. No ( )

Q6. Which groups do you belong to?
1. Church group ( )
2. Community group ( )
3. Work place group ( )
4. Other (please specify)…………………………

Q7. Did you receive gifts before the birth of your child?
1. Yes ( )
0. No ( )

Q8. If yes, what did you receive?
1. Money ( )
2. Foodstuff ( )
3. Toiletries (washing soap, diapers, cosmetics) ( )
4. Other (please specify)……………………..

Q9. What monetary value will you attach to the gifts you received?

Section D: Birth Preparedness

Q1. Do you know about birth preparedness?
1. Yes ( )
0. No ( )

Q2. What is birth preparedness?
1. Preparations made towards birth ( )
2. Preparations made towards pregnancy ( )
3. Preparations for marriage ( )

Q3. How did you get to know about it?
1. Antenatal Clinic ( )

2. Media ( )
3. Friends ( )
4. Family ( )

7- Item birth preparedness checklist (MCHR, 2018)

Q1. Did you make arrangements for transport towards delivery?
1. Yes ( )
0. No ( )

Q2. Did you arrange for helpers to take care of the home whiles you are away?
1. Yes ( )
0. No ( )

Q3. Did you decide on the facility you intended to deliver?
1. Yes ( )
0. No ( )

Q4. Did you save money towards delivery?
1. Yes ( ) How much? ……………
0. No ( )

Q5. Did you have a valid health insurance card?
1. Yes ( )
0. No ( )

Q6. Did you prepare for a blood donor?
1. Yes ( )
0. No ( )

Q7. Did you know about any of the following danger signs?

Question	Response Options
1. Headache	1. Yes ( ) 0. No ( )
2. Swollen feet, arms and face	1. Yes ( ) 0. No ( )
3. Convulsive fits	1. Yes ( ) 0. No ( )
4. The bag of water breaks before the expected date of delivery	1. Yes ( ) 0. No ( )

5. Dizziness, difficulty in breathing and rapid heart beating — 1. Yes ( ) 0. No ( )

6. Increase in body temperature — 1. Yes ( ) 0. No ( )

7. Increase or decrease or no movement of your baby — 1. Yes ( ) 0. No ( )

8. Smelly and or greenish water from the birth canal — 1. Yes ( ) 0. No ( )

9. Persistent vomiting — 1. Yes ( ) 0. No ( )

10. Severe abdominal pain — 1. Yes ( ) 0. No ( )

11. Bleeding — 1. Yes ( ) 0. No ( )

Wealth Index

1. Do you own a flat screen television? 1. Yes ( ) 0. No ( )
2. Does any member of this household own any land? 1. Yes ( ) 0. No ( )
3. A refrigerator? 1. Yes ( ) 0. No ( )
4. Home theater? 1. Yes ( ) 0. No ( )
5. Does any member of this household have a bank account? 1. Yes ( ) 0. No ( )
6. Electricity? 1. Yes ( ) 0. No ( )
7. Do you own a vehicle? 1. Yes ( ) 0. No ( )
8. Do you own a room divider? 1. Yes ( ) 0. No ( )
9. What type of fuel does your household **mainly** use for cooking?
1. LPG ( )
2. Wood ( )
3. Other ( )
10. What kind of toilet facility do members of your household **usually** use?
1. Water closet ( )
2. KVIP (Public) ( )
3. KVIP (Private) ( )
4. bush / field ( )
11. What is the **main** source of drinking water for members of your household?
1. Satchet water ( )
2. Bottle water ( )
3. Pipe water ( )
12. What is the **main** material of the floor in your household?

1. Cement ( )
2. Tiles ( )
3. Terazo ( )
4. Bareground ( )

13. What is the **main** material of the ceiling in your household?

1. POP( )
2. Wood ( )
3. Cement ( )
